# Multilevel-analysis identify a *cis*-expression quantitative trait locus associated with risk of renal cell carcinoma

**DOI:** 10.18632/oncotarget.3001

**Published:** 2015-02-25

**Authors:** Xiang Shu, Mark P. Purdue, Yuanqing Ye, Christopher G. Wood, Meng Chen, Zhaoming Wang, Demetrius Albanes, Xia Pu, Maosheng Huang, Victoria L. Stevens, W. Ryan Diver, Susan M. Gapstur, Jarmo Virtamo, Wong-Ho Chow, Nizar M. Tannir, Colin P. Dinney, Nathaniel Rothman, Stephen J. Chanock, Xifeng Wu

**Affiliations:** ^1^ Department of Epidemiology, The University of Texas MD Anderson Cancer Center, Houston, Texas, USA; ^2^ Division of Cancer Epidemiology and Genetics, National Cancer Institute, National Institutes of Health, Department of Health and Human Services, Bethesda, Maryland, USA; ^3^ Urology, The University of Texas MD Anderson Cancer Center, Houston, Texas, USA; ^4^ Cancer Genomics Research Laboratory, SAIC-Frederick Inc., National Cancer Institute-Frederick, Frederick, Maryland, USA; ^5^ Epidemiology Research Program, American Cancer Society, Atlanta, Georgia, USA; ^6^ Department of Chronic Disease Prevention, National Institute for Health and Welfare, Helsinki, Finland; ^7^ Genitourinary Medical Oncology, The University of Texas MD Anderson Cancer Center, Houston, Texas, USA

**Keywords:** RCC, GWAS, GSEA, eQTL

## Abstract

We conducted multilevel analyses to identify potential susceptibility loci for renal cell carcinoma (RCC), which may be overlooked in traditional genome-wide association studies (GWAS). A gene set enrichment analysis was performed utilizing a GWAS dataset comprised of 894 RCC cases and 1,516 controls using GenGen, SNP ratio test, and ALIGATOR. The antigen processing and presentation pathway was consistently significant (*P* = 0.001, = 0.004, and < 0.001, respectively). Versatile gene-based association study approach was applied to the top-ranked pathway and identified the driven genes. By comparing the expression of the genes in RCC tumor and adjacent normal tissues, we observed significant overexpression of *HLA* genes in tumor tissues, which was also supported by public databases. We sought to validate genetic variants in antigen processing and presentation pathway in an independent GWAS dataset comprised of 1,311 RCC cases and 3,424 control subjects from the National Cancer Institute; one SNP, rs1063355, was significant in both populations (P_meta-analysis_ = 9.15 × 10^−4^, *P*_heterogeneity_ = 0.427). Strong correlation indicated that rs1063355 was a *cis*-expression quantitative trait loci which associated with *HLA-DQB1* expression (Spearman's rank *r* = −0.59, *p* = 5.61 × 10^−6^). The correlation was further validated using a public dataset. Our results highlighted the role of immune-related pathway and genes in the etiology of RCC.

## INTRODUCTION

Renal cell carcinoma (RCC) accounts for more than 80% of kidney cancers [1]. The incidence of kidney cancer has been increasing since the 1970s [2], and the disease is among the top 10 most common cancers for both males and females in the United States [2, 3]. Cigarette smoking, obesity, and hypertension are well-known modifiable risk factors for RCC [2]. Other epidemiological risk factors include red meat consumption and occupational exposure to trichloroethylene [4], whereas alcohol, fruit, and vegetable consumption is suspected to be protective [2].

Genetic susceptibility also plays an important role in RCC risk. Individuals who have a first-degree relative with a history of kidney cancer are at more than twice the risk for developing RCC [5, 6]. A handful of genes, such as *VHL* and *FH*, explain a fraction of the known inherited kidney cancer syndromes [7–11]. The candidate gene approach identified several genes that could be involved in the development of sporadic RCC, such as *MET*, *KILLIN*, and *FLCN* [12–14]. In recent years, three susceptibility loci at the following chromosomal regions, 2p21 (*EPAS1)* [15], 11q13 (a *CCND1* transcriptional-enhancer site) [15–17] and 2q22.3 (*ZEB2)* [18], have been identified for RCC by genome-wide association studies (GWAS). In addition, we previously identified a common genetic variant at 12p11 (*ITPR2)* that was associated with RCC risk [16]. The locus, which was also identified by GWAS to be associated with waist-to-hip ratio [19], may provide insight into the relationship between obesity and RCC etiology.

Although GWAS and meta-analyses conducted by large consortia have been successful in identifying SNPs associated with complex diseases, most of these SNPs are located in intergenic regions and their biological mechanisms are largely unknown. A stringent criterion for significance (*P* < 5 × 10^−8^) of GWAS findings in order to reduce false positive results due to multiple testing is widely accepted. In contrast, the use of gene- and pathway-based analyses of GWAS data, which takes into account the aggregated effects within a gene or pathway, substantially reduces the multiple testing burden by combining numerous genes and pathways into a reduced number of gene sets. In this study, we searched for novel potential RCC genetic susceptibility loci through analyses in pathway, gene and SNP levels, using RCC GWAS data, gene expression data, copy number variation data, public datasets and online resources.

## RESULTS

Twenty one pathways were consistently significant with *p* < 0.05 for all three algorithms (Table 1). In our analysis, most of the pathways identified were either immune- or cancer-related. The coverage of genes tagged by GWAS SNPs for each pathway was 80% or higher. However, only the antigen processing and presentation pathway remained significant after multiple comparison correction with a false discovery rate of 0.20. We adjusted the top 10 principal components to control the population substructure. Results of sensitivity analysis showed the antigen processing and presentation pathway was a promising candidate (Table S1).

**Table 1:** Significant^1^ pathways identified by GenGen, SNP ratio test, and ALIGATOR

Databases and pathways	Number of genes in pathway given by databases	Number (%) of genes tagged by study GWAS SNPs	*P* value ^2^		
			GenGen	SNP ratio test	ALIGATOR
**KEGG**					
Antigen processing and presentation	89	78 (87.6%)	0.001 (0.104)	0.004 (0.122)	< 0.001 (0.028)
Asthma	30	26 (86.7%)	0.019 (0.423)	0.027 (0.167)	0.043 (0.986)
Allograft rejection	38	33 (86.8%)	0.007 (0.710)	0.013 (0.167)	0.006 (0.446)
Graft versus host disease	42	34 (81.0%)	0.015 (0.385)	0.030 (0.167)	0.027 (0.922)
Intestinal immune network for IGA production	48	44 (91.7%)	0.021 (0.635)	0.030 (0.167)	0.003 (0.246)
JAK STAT signaling	155	146 (94.0%)	0.033 (0.335)	0.002( 0.122)	0.023 (0.889)
Leishmania infection	72	63 (87.5%)	0.013 (0.571)	0.029 (0.167)	0.026 (0.916)
Nod like receptor signaling pathway	62	58 (93.5%)	0.016 (0.472)	0.049 (0.167)	0.016 (0.777)
T cell receptor signaling pathway	108	104 (96.3%)	0.019 (0.467)	0.016 (0.196)	0.015 (0.762)
**BioCarta**					
Cytokine	22	21 (95.5%)	0.003 (0.338)	< 0.001 (0.134)	0.017 (0.720)
DC	22	21 (95.5%)	< 0.001 (0.151)	0.002 (0.134)	0.011 (0.600)
**Reactome**					
CREB phosphorylation through the activation of RAS	27	23 (85.2%)	0.039 (0.850)	0.006 (0.408)	0.005 (0.509)
CREB phosphorylation through the activation of CAMKII	15	15 (100%)	0.047 (0.781)	0.011 (0.523)	0.006 (0.600)
NEF mediates down modulation of cell surface receptors by recruiting them to clathrin adapters	21	20 (95.2%)	0.049 (0.810)	0.026 (0.586)	0.015 (0.877)
**GO**					
Microtubule cytoskeleton	152	138 (90.8%)	0.007 (0.462)	0.048 (0.528)	0.013 (0.995)
Hematopoietin interferon classd200 domain cytokine receptor binding	29	29 (100%)	0.007 (0.712)	0.020 (0.528)	0.013 (0.995)
Cytokine activity	113	108 (95.6%)	0.010 (0.413)	0.013 (0.528)	0.015 (0.998)
Response to temperature stimulus	16	15 (93.8%)	0.025 (0.813)	0.048 (0.528)	0.007 (0.961)
Negative regulation of transferase activity	35	34 (97.1%)	0.025 (0.720)	0.007 (0.528)	0.022 (1.000)
Kinase regulator activity	46	43 (93.5%)	0.030 (0.690)	0.016 (0.528)	0.014 (0.998)
Positive regulation of t cell proliferation	13	12 (92.3%)	0.037 (0.934)	0.031 (0.528)	0.019 (1.000)

1 Significance was determined based on *p* < 0.05 calculated by all three tests.

2 Values in parentheses are FDR corrected.

We further investigated the genes that drove the association for the antigen processing and presentation pathway. VEGAS results revealed that eight genes belonging to the pathway were significantly associated with RCC risk (Table 2). Four genes belong to the HLA family: *HLA-DQA1*, *HLA-DRB1*, *HLA-DQB1*, and *HLA-F*, which were significantly overexpressed in RCC tumor tissues compared with paired adjacent normal tissues (Table 2). *CREB1* and *CTSL1* were slightly overexpressed but not statistically significant, while *PSME3* showed a significantly reduced level in RCC tumor tissues. Among them, *HLA-DQB1* had the greatest difference between paired tumor and normal tissue with a 21% increased expression in RCC tumor tissues. In addition, our findings were supported by TCGA data and 6 datasets available in Oncomine. The upregulation of HLA genes between paired tumor and normal tissue were robust in all datasets (Figure 2, Table S2 and Figure S2). No chromosomal alteration was observed for 6p21.3 in our Array Comparative Genomic Hybridization (array-CGH) data, where HLA genes are located (data not shown), and no copy number variation was observed in TCGA dataset implemented in Oncomine (data not shown). Furthermore, no somatic mutations of HLA genes were found in RCC tissue according to COSMIC database (data not shown).

**Figure 1:**
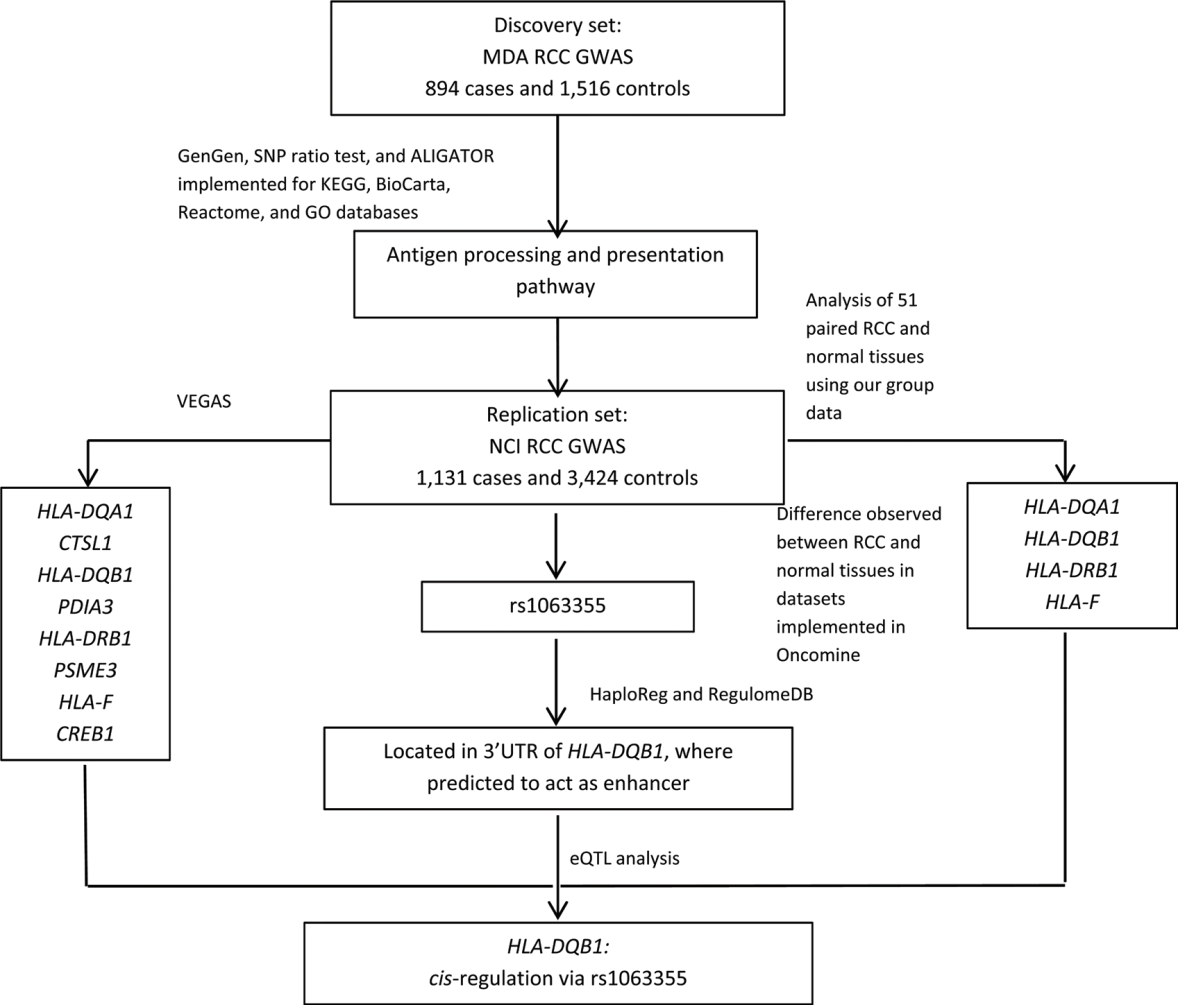
Study flowchart

**Figure 2:**
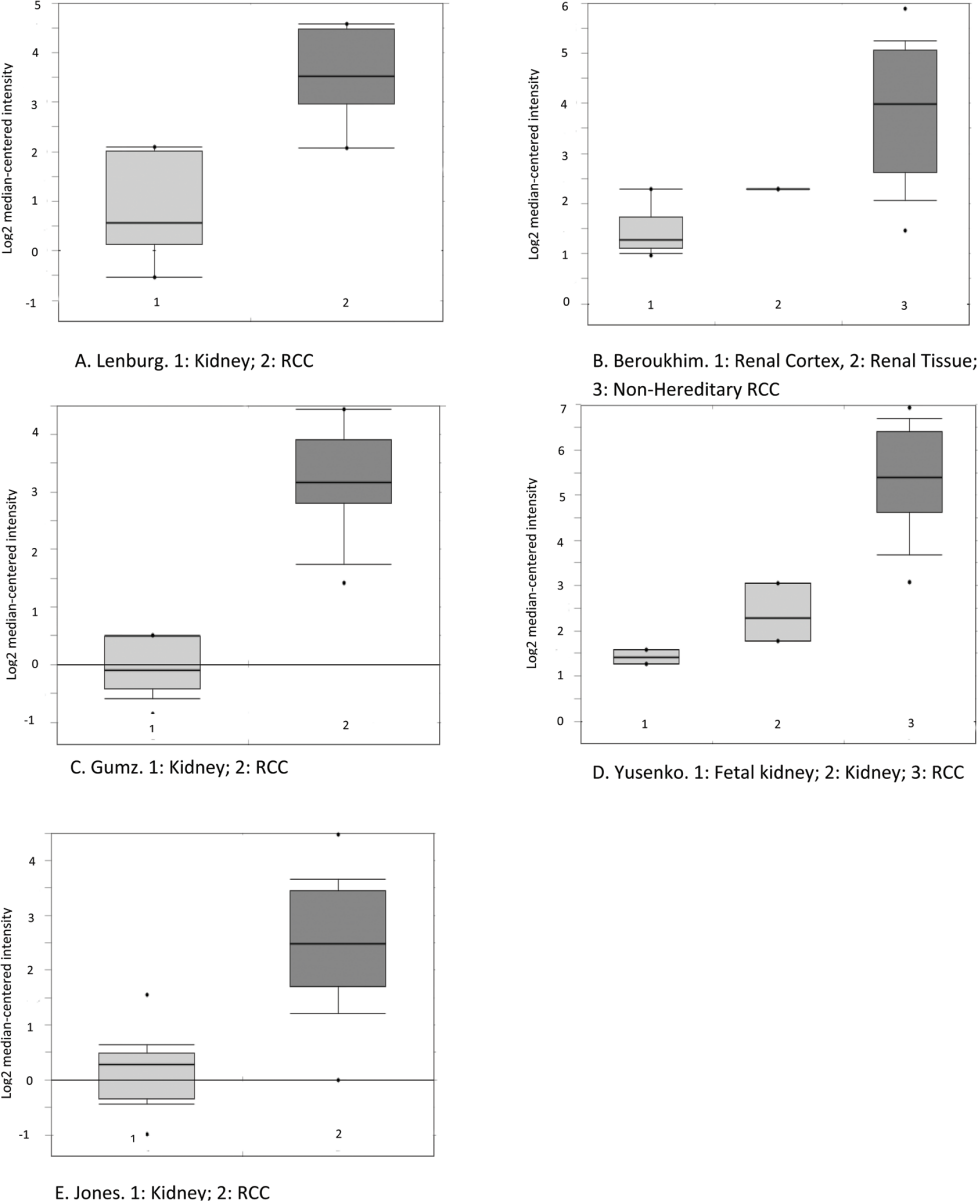
Boxplot of *HLA-DQB1* mRNA levels in RCC and adjacent normal tissue as reported in five datasets available in the Oncomine database The probe selected for all datasets (212998_×_at) was defined in Oncomine. (The sixth study was not included here because its platform was not pre-defined in Oncomine, although the change in the same direction was detected.) A, Lenburg. B, Beroukhim. C, Gumz. D, Yusenko. E, Jones. Circles stand for outliers. The figures were directly downloaded from Oncomine.

**Table 2:** VEGAS gene-based test results of the antigen processing and presentation pathway and gene expression comparison between paired RCC and adjacent normal tissues

				Gene expression level^2^ (mean ± SD)			
Gene	Chromosome	No. of SNPs mapped to gene	P_VEGAS_^1^	Normal tissue	RCCtissue	Fold change^3^	*P* value^4^
HLA-DQA1	6	11	0.0039(0.094)	9.30(1.31)	10.91(1.48)	1.17	3.92E-08
CTSL1	9	16	0.0048(0.094)	10.09(0.79)	10.40(1.11)	1.03	0.076
HLA-DRB1	6	8	0.0051(0.094)	8.24(2.10)	9.04(2.55)	1.10	5.61E-04
HLA-DQB1	6	7	0.0082(0.113)	7.20(1.17)	8.69(1.72)	1.21	5.00E-09
PDIA3	15	2	0.0120(0.133)	N.A.	N.A.	N.A.	N.A.
PSME3	17	1	0.0181(0.167)	8.60(0.70)	8.16(0.70)	0.95	2.59E-06
HLA-F	6	25	0.0378(0.299)	8.18(0.97)	9.68(1.15)	1.18	2.67E-11
CREB1	2	9	0.0436(0.299)	7.00(0.53)	7.14(0.59)	1.02	0.103

*HLA-DQA1*: major histocompatibility complex, class II, DQ alpha 1.

*CTSL1*: cathepsin L1.

*HLA-DRB1*: major histocompatibility complex, class II, DR beta 1.

*HLA-DQB1*: major histocompatibility complex, class II, DQ beta 1.

*PDIA3*: protein disulfide isomerase family A, member 3.

*PSME3*: proteasome activator subunit 3.

*HLA-F*: major histocompatibility complex, class I, F.

*CREB1*: cAMP responsive element binding protein 1.

1 P_VEGAS_ was obtained using VEGAS, corresponding *q* value was listed in the parenthesis.

2 Expression data were quantile normalized and log2 transformed.

3 Fold change = RCC/Normal, based on mean of log2 transformed data.

4 *P* value was calculated by paired Student's *t*-test.

N.A.: Data not available.

We sought to validate SNPs located in our top significant pathway in an independent population. After filtering SNPs in strong linkage disequilibrium (R^2^ > 0.8), 48 significant SNPs (all *p* < 0.05) in antigen processing and presentation pathway were sent to NCI (Table S3) for in silico validation. Only one SNP, rs1063355, was significantly associated with RCC risk in both the MD Anderson GWAS and the NCI GWAS (Table 3). The minor allele frequency of rs10663355 in control subjects was similar in two populations. Possessing one A allele of rs1063355 increased by 10%–20% the risk of RCC in both MD Anderson and NCI populations (*P* = 0.007 and 0.039, respectively). The odds ratio for the combined MD Anderson and NCI data using fixed-effect meta-analysis was 1.14 (*P* = 9.15 × 10^−4^, Cochran's Q test, I^2^: 0.0%, *P*_heterogeneity_ = 0.427). We imputed SNPs within +/−1 Mb of rs1063355 (Figure S3).

**Table 3:** Validation of SNPs in antigen process and presentation pathway

SNP	Nearby Gene	Minor	MAF^§^	OR (95%CI)^¶^	*P* value	Higgins' I^2^	*P*_heterogeneity_
rs1063355	HLA-DQB1						
MDA		A	0.44/0.40	1.19 (1.05–1.34)	0.007		
NCI		A	0.44/0.43	1.11 (1.01–1.23)	0.039		
Overall				1.14 (1.06–1.23)	9.15E-4^&^	0.0%	0.427

§ MAF: minor allele frequency in cases/control.

¶ Adjusted for age (5-year intervals), and sex under additive model.

& Meta *p*-value is calculated assuming fixed effect model.

The SNP rs1063355 is located in the 3′-untranslated region of *HLA-DQB1*. ENCyclopedia Of DNA Elements (ENCODE) data showed that rs1063355 is located within the area predicted to act as enhancers in HepG2 and GM12878 cell lines (Table S4), with four proteins (e.g. TBP, ELF1, EBF1, and TCF12) bounding to the region. The details were also visually available in the figure downloaded from the UCSC Genome Browser (Figure S4). Interestingly, the SNP was found to be in expression quantitative trait locus (eQTL) with *HLA-DQB1*.

**Figure 3:**
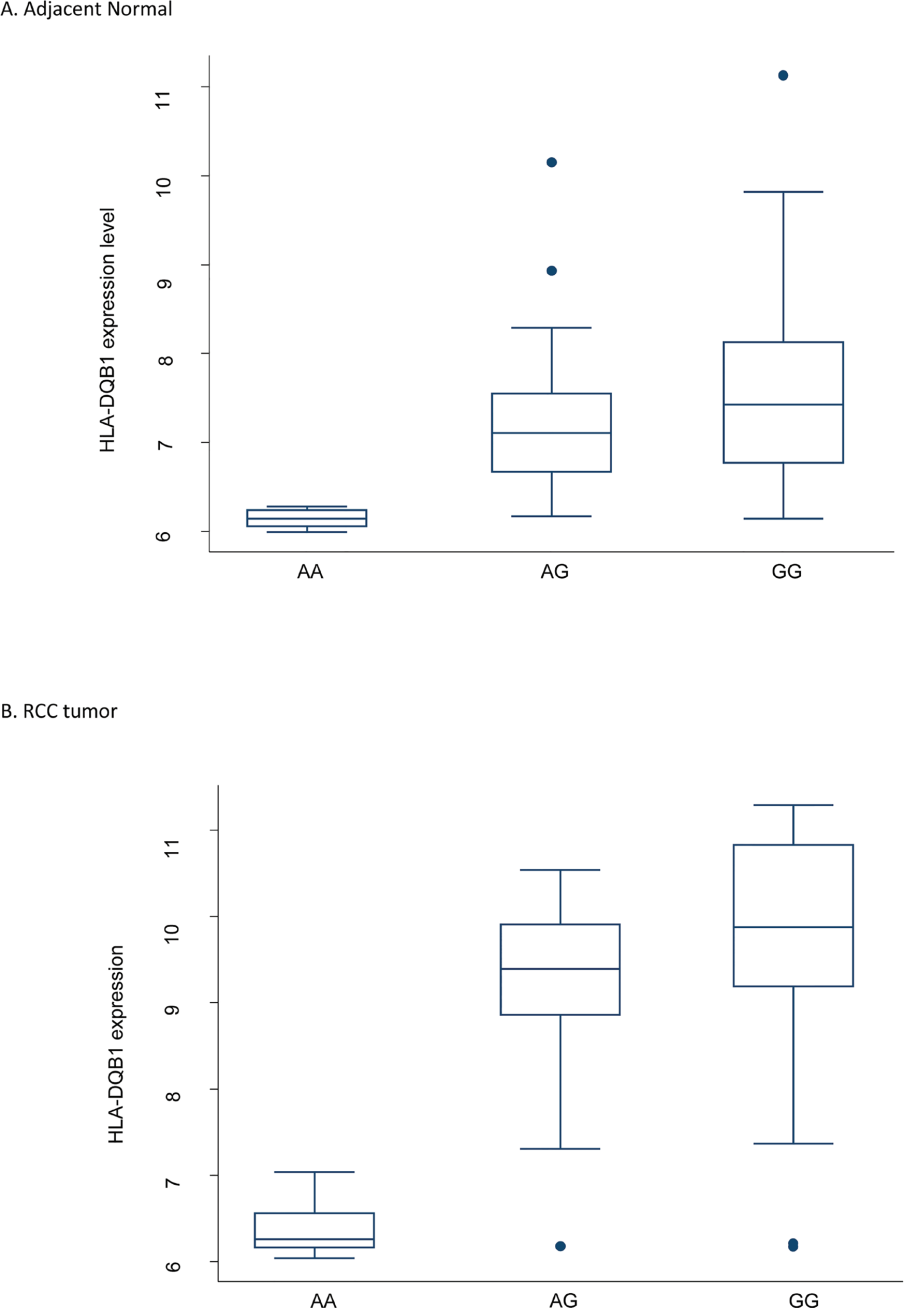
Boxplot of *HLA-DQB1* mRNA level categorized by rs1063355 genotype Both genotyping and gene expression data were available 51 pairs of RCC and adjacent normal tissues collected at MD Anderson. The genotype was CC for 18 study subjects, AC for 22, and AA for 11. Spearman's *r* = −0.59, P_trend_ = 5.61E-6 in normal tissue. The same trend was observed in tumor tissue. The coefficient obtained from simple linear regression was ™0.80 (95% CI = −1.18 to −0.41, *p* < 0.001). The expression level of *HLA-DQB1* was log2 transformed. Circles stand for outliers.

**Figure 4:**
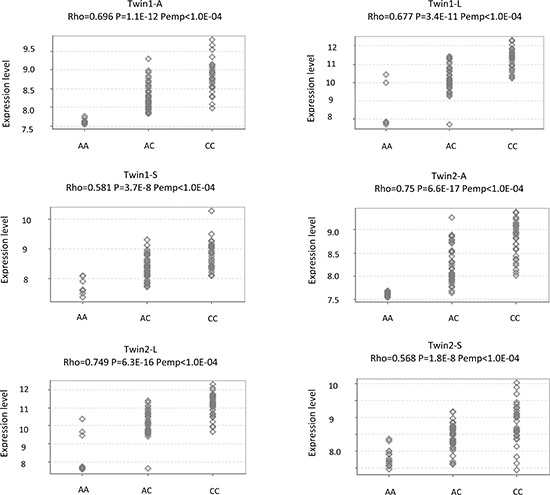
eQTL analysis for rs1063355 and *HLA-DQB1* in a public dataset The MuTHER pilot study collected adipose tissue (A), lymphoblastoid cell lines (L), and skin tissue (S) from healthy Caucasian female twins. All figures were directly downloaded from Genevar. Rho: Spearman's correlation coefficient. P: Corresponding *p* value. Pemp: Empirical *p* values calculated from 10,000 permutations.

To further explore the SNP-gene relationship, we performed cis-eQTL analysis for rs1063355 in *HLA-DQB1* in 51 paired RCC and adjacent normal tissues collected by our group. There were 18, 22, and 11 patients with CC, AC, and AA genotypes, respectively. The minor allele of rs1063355 (risk allele A) was associated with lower log2 transformed *HLA-DQB1* mRNA level in adjacent normal tissues (Figure 3). Spearman's rank correlation coefficient was −0.59 (*p* = 5.61 × 10^−6^). Result of linear model showed possessing one copy of risk allele of rs1063355 could reduce 0.80 unit of log2 transformed *HLA-DQB1* mRNA level (*p* < 0.001, data not shown). The same trend was observed in RCC tumor tissue (data not shown). We further evaluated the correlation for all SNPs in 3′UTR of *HLA-DQB1* where rs1063355 is located. Rs1063355 was in high LD with the imputed SNP which possessed the top significant GWAS and eQTL association (e.g. rs1063345, R^2^ = 0.99) in both tumor and normal tissues (Table S5). To corroborate these findings, we used public MuTHER dataset for replication. It supported our findings that *HLA-DQB1* was under-expressed in lymphoblastoid cell lines, adipose tissues, and skin tissues of subjects with the AA genotype of rs1063355 compared to subjects with the AC or CC genotype. The correlations remained significant in permutation test for three types of tissue (Figure 4).

## DISCUSSION

To our knowledge, this is the first study to use multilevel approaches including discovery analysis of GWAS, gene expression correlation analyses, and online resources to investigate the aggregated effect of common SNPs in relation to RCC etiology with respect to defined pathways and genes. To assure reliability, three analytical algorithms and four commonly used pathway collection databases were applied in pathway analysis with correction for multiple comparisons. Our identification of the antigen processing and presentation pathway and *HLA* genes supports an important role for the immune system in RCC etiology. The validation on SNP level and eQTL analysis identified a new potential susceptibility region and a putative functional SNP which could help to elucidate the biological mechanisms underlying RCC development.

The results of VEGAS and the gene expression level comparison between RCC tumor and adjacent normal tissues indicated that major histocompatibility complex (MHC) loci, in particular *HLA-DQB1,* may contribute to RCC etiology. *HLA-DQB1* belongs to HLA class II beta chain paralogs which, along with an alpha chain, forms the HLA class II heterodimer. The HLA-DQ protein is usually expressed on the surface of antigen presenting cells and plays a critical role in preparing and presenting peptides to T cells. The difference in gene expression levels could be related to local copy number variation. However, we did not find any alteration in the region in either our own data or in TCGA data, indicating that the expression may be affected through other mechanisms. Considering the location of rs1063355, we hypothesized that this SNP or linked SNPs were associated with the expression level of *HLA-DQB1*.

Interestingly, the contrasting results of associations between rs1063355, *HLA-DQB1* expression, and RCC risk suggested a complex relationship. Since the risk variant (allele A) of rs1063355 were associated with reduced *HLA-DQB1* expression, our results suggested that underexpression of *HLA-DQB1* may increase the RCC risk. In contrast, overexpression of *HLA-DQB1* found in RCC tissue revealed the complexity of abnormal alterations in tumor tissue. The inflammation that occurs during cancer development may actually induce MHC expression in tissues or tumor cells [20, 21], which may support the observation of higher expression level of *HLA-DQB1* in RCC tissues. Thus, we hypothesized that reduced *HLA-DQB1* expression may play a crucial role to avoid immune surveillance during tumorigenesis, but overexpression may be an adaptive response once the transformation is complete. Future functional assays are needed to elucidate this sophisticated framework.

Regions on or close to *HLA-DQB1* (6p21.3) were frequently identified by GWAS as susceptibility loci for many complex diseases, such as lymphoma [22], type 1 diabetes [23], asthma [24], systemic sclerosis [25], and narcolepsy [26]. The intergenic region between *HLA-DQB1* and *HLA-DQB2* was linked to IgA nephropathy in one GWAS study [27]; but to date no epidemiologic study has linked IgA nephropathy to kidney malignancy. In addition, one study has reported that multi-loci haplotypes were associated with a risk for cervical cancer [28]. The region was also found to be associated with hepatitis B virus–related hepatocellular carcinoma risk in Chinese population [29].

The present study has numerous strengths, including large sample sizes for the discovery and replication populations. Additionally, we were able to validate the finding at the SNP and gene expression level. We were also able to validate the eQTL analyses by using a publicly available dataset. Importantly, the SNPs we identified may regulate *HLA-DQB1* transcription level *in cis*. However, limitations of this study warrant consideration. Specifically, the biological mechanism that describes how this SNP affects *HLA-DQB1* expression was not investigated and remains unknown. It is possible that other linked functional SNPs, rather than rs1063355, contribute to the difference in *HLA-DQB1* expression level observed in individuals with distinct genotypes. In addition, the identified pathway and genes showed modest significance when multiple testing is considered, and only one SNP in antigen processing and presentation pathway was significant in NCI samples with moderate *p* value. Nevertheless, there is biological plausibility for the association of *HLA-DQB1* and cancer risk. The evidence that rs1063355 or other SNPs in linkage disequilibrium could be potentially functional and driving the association, is promising. Thus, the locus remains interesting to be further investigated.

In conclusion, the results of multilevel analyses in this study support the idea that the HLA class II region may influence RCC tumorigenesis. Moreover, we found a variant in *HLA-DQB1*, replicated in an independent population, could alter cancer risk in a cis-eQTL manner. However, overexpression of *HLA-DQB1* in RCC tissue revealed the complexity of the biological mechanisms underlying the process of tumor formation. Further studies are required to validate our findings. Functional assays are needed to elucidate the biological mechanism involved in the regulation of *HLA-DQB1* expression and the SNP's role in RCC etiology.

## MATERIALS AND METHODS

Figure 1 illustrates the steps used in this study to identify potential susceptibility loci for RCC.

### Study population

The details of the study population for the RCC GWAS conducted previously have been described elsewhere [16]. Briefly, newly diagnosed and histologically confirmed RCC cases and healthy control subjects were recruited from an ongoing RCC case-control study that began in 2002 at The University of Texas MD Anderson Cancer Center in Houston, TX. The recruitment of control subjects in Texas was performed via random digital dialing [30]. An additional set of control subjects from an ongoing bladder cancer case-control study, who were involved in a previously published GWAS of bladder cancer, was also included [31]. Recruitment was not restricted by age, sex, ethnicity, or cancer stage. A control subject had to have lived for no less than 1 year in the same county or socio-economically matched surrounding counties where a case subject resided. Healthy controls were individuals who had no history of cancer (except non-melanoma skin cancer) at the time of recruitment. Cases and controls were frequency matched by age (± 5 years), sex, and county of residence. However, only cases and controls who self-reported to have European ancestry were included in the analysis of our RCC GWAS study. Informed consent had been obtained from all study participants before epidemiological data and blood samples were collected by trained MD Anderson staff interviewers. The study was approved by the Institutional Review Board at the MD Anderson Cancer Center, and informed consent was obtained from all participants for discovery set.

### Validation population

We used the U.S. National Cancer Institute (NCI) RCC GWAS to validate statistically significant SNPs (*p* < 0.05) identified from the MD Anderson GWAS. The NCI participants had been recruited from 4 studies [Prostate, Lung, Colorectal and Ovarian Cancer Screening Trial (PLCO), American Cancer Society Cancer Prevention Study II Nutrition Cohort (CPS-II), Alpha-Tocopherol, Beta-Carotene Cancer Prevention Study (ATBC), and National Cancer Institute United States Kidney Cancer Study (USKC)] and informed consent had been collected from each participant. After the quality control procedures were completed, the study comprised of 1,311 cases and 3,424 controls. The details of the study design and population characteristics were previously described [15]. Informed consent was obtained from all participants, and each study was approved by the appropriate institutional review boards and/or ethics committees for replication set.

### Genotyping

Information on the platforms used for the primary scan of our population and the quality controls were described previously [16]. In short, the primary scan for the discovery population was performed at MD Anderson using HumanHap610/660W BeadChips (Illumina, San Diego, CA, USA) [16, 31]. After quality control procedures were completed, 2,410 samples, including 894 RCC cases and 1,516 healthy controls were available for analysis. A total of 533,191 SNPs were included in the final analysis. There was no evidence of differences in population substructure (inflation factor λ = 1.037). HumanHap 500, 610, or 660W BeadChips were used in the primary scan of the NCI population; details can be found in a previous publication [15].

### Gene expression, eQTL analysis, copy number variation, and mutation spectrum in tissues

Gene expression assays were performed in 51 pairs of RCC tumor tissue and adjacent normal tissue collected from patients who had been recruited to our RCC case-control study. Total RNA was isolated using the mirVana RNA isolation kit (Ambion, Austin, TX) according to the standard protocol from approximately 20 mg of flash-frozen tissue, which was placed in RNAlater-ICE frozen tissue transition solution (Ambion) at −20°C. HumanHT-12 v2 Expression BeadChip kits (Illumina) was used to profile the whole genome-wide gene expression and were read using a BeadStation 500 scanner (Illumina). Arrays were quantile normalized and the data were log2 transformed. To corroborate our results, we also checked genes from top significant pathway in Oncomine. Six studies were available for the analysis in Oncomine [32–37]. For robustness of each gene, we compared the number of studies with significantly altered expression level, average *p*-value, and median rank (sort by *p*-value) among genes across all datasets in Oncomine. We also used USCS Cancer Browser to explore the gene expression level for our genes in the TCGA database.

We conducted expression quantitative trait loci (eQTL) analysis for our candidate SNP using mRNA microarray data generated from paired RCC tumor and adjacent normal tissues. To show rs1063355 was the best GWAS and eQTL SNP in the region, we also assessed the correlation for all imputed and genotyped SNPs physically close to it (chromosomal region of 3′UTR of *HLA-DQB1*). The public resource Genevar [38] contains 4 eQTL studies which could be used for the replication set. However, only the MuTHER pilot study [39] has both rs1063355 genotyping and *HLA-DQB1* expression data available for the analysis. Three types of tissues were collected including lymphoblastoid cell lines, adiposity tissues, and skin tissues in the MuTHER pilot study. We checked the Spearman's correlation of SNP-gene within a 1 Mb region where the SNPs is located for all three types of tissue.

We checked the copy number variation in the region identified using the data produced by our group using a method described previously [40]. TCGA Renal 2 data implemented in Oncomine was also used for assessing the gene copy number variation. It compared copy number of genes among 489 clear cell renal cell carcinoma, 43 papillary renal cell carcinoma, 441 paired normal kidney tissue samples and 98 paired normal blood specimen.

Information on the somatic mutations of significant genes identified in our analyses can be found in the Catalogue of Somatic Mutations in Cancer (COSMIC).

### SNP function annotations

To predict the putative function of rs1063355, we used HaploReg [41] and RegulomeDB [42] to analyze the ENCODE data [43].

### Statistical analysis

We applied three gene set enrichment analysis (GSEA) tools to four well-characterized pathway databases. Gene-based tests were performed for the most promising pathway we identified. Gene expression levels were compared between paired RCC and adjacent normal tissue. We sought validation from SNP level in an independent NCI RCC GWAS.

#### Pathway Databases

Four frequently used pathway databases (KEGG, BioCarta, Reactome, and GO) were downloaded from the Molecular Signatures Database by selecting “C2: Curated gene sets” (for KEGG, BioCarta, and Reactome) or “C5: GO gene sets” (for GO). Three types of datasets were available from the Molecular Signatures Database; we used the file contained “Entrez Gene IDs”. This dataset contained 186, 217, 430 and 1454 gene sets in KEGG, BioCarta, Reactome, and GO, respectively.

#### Gene Annotation

We used the gene annotation file “NCBI Build 36” from the National Center for Biotechnology Information website. This file provided gene location information.

#### SNP Mapping to Genes

We used the University of California, Santa Cruz Genome Browser to retrieve the locus information for each SNP of interest by selecting “NCBI 36/hg 18” and “SNP 129”. A total of 533,126 SNPs was matched with the database and their positions in a specific chromosome were successfully obtained. We restricted our analysis to autosomal chromosomes, such that 12,440 SNPs in chromosome X and 17 SNPs in chromosome Y were removed from the analysis. Thus, 520,669 SNPs remained to be mapped to specific genes. SNPs within 20 kb upstream or downstream of a gene were considered to belong to that gene; some SNPs were mapped to more than one gene because of overlapping sequences. Due to the design of the array, not all the genes located in a pathway were captured by our GWAS data.

#### GSEA Tools

GenGen [44]. The methodology of GenGen has been described previously. The concept was inspired by GSEA for microarray data. In this approach, an enrichment score is calculated. One thousand permutations are performed, and the permutation-based (1,000-time) false discovery rate is calculated to assess the issue of multiple comparisons.

SNP Ratio Test [45]. This test calculates the proportion of significant SNPs in a specified pathway. The empirical *p*-value is calculated based on 1,000 permutations. We also calculated the false discovery rate for empirical *p*-values.

ALIGATOR [46]. This approach counts the number of significant genes represented by significant SNPs. Each significant gene is counted only once regardless of how many significant SNPs map to that gene. A bootstrap approach (repeated 1,000 times) is applied to correct the empirical *p*-values.

In addition, we adjusted the size of the pathway by confining the number of genes to between 10 and 200. Finally, 180, 206, 402 and 1,226 pathways were included in the analysis for the KEGG, BioCarta, Reactome, and GO databases, respectively. A pathway was considered significant if the *p*-value was < 0.05 for all three GSEA algorithms. We further adjusted for top 10 principal components (Figure S1) in the model and performed the same analyses. Principal component analysis was conducted using EIGENSTRAT [47].

#### Versatile Gene-Based Association Study (VEGAS)

For the gene-based test, we used VEGAS [48] to investigate aggregated signals within specific genes located in the most promising pathway. SNP level *p*-values were used as input into the program to produce an empirical gene-based *p*-value by simulation.

#### Validation of SNPs in a Pathway

We extracted all SNPs mapped to the most promising pathway. Multivariable logistic regression adjusted for age (in 5-year intervals) and sex were conducted for each SNP in an additive model. We sought to replicate only significant SNPs (*p* < 0.05). From a subset of SNPs located in the same linkage disequilibrium block (R^2^ > 0.8), the SNP with the smallest *p* value was selected to represent the block. Finally, 48 SNPs were selected for validation with the NCI RCC GWAS population. Results of two studies were pooled by meta-analysis. Selection of a fixed-effect model depended on the results of Cochran's Q test for heterogeneity being *P*_heterogeneity_ ≥ 0.05; otherwise, a random-effect model would be adopted. The per-allele trend effect was estimated and the *P* value was computed using inverse variance weighting.

#### Other Statistical Analyses

For data generated from samples collected by our group, paired *t* test was used to compare gene expression between paired RCC and adjacent normal tissues. Spearman's rank correlation, linear model and corresponding *p*-values were calculated for SNPs and genes of interest. Imputation of the +/−1 Mb region of where a SNP was located was conducted with IMPUTE2 [49, 50]. After quality control, 10,909 SNPs (Imputation Score ≥ 0.5, MAF > 0.01, HWE *p*-value < 0.001, genotype call rate > 0.90) were found in *a* ± 1 Mb region up/downstream of where rs1063355 is located. All statistical analyses were done using Stata 10.0 (College Station, TX, USA). All correlation association analyses were conducted with respect to the minor allele.

### Web resources

Oncomine: https://www.oncomine.org

USCS Cancer Browser: https://genome-cancer.ucsc.edu/proj/site/hgHeatmap/

Genome Browser: http://genome.ucsc.edu/

COSMIC: http://cancer.sanger.ac.uk/cancergenome/projects/cosmic/

Molecular Signatures Database: http://www.broadinstitute.org/gsea/msigdb/index.jsp

NCBI: http://www.ncbi.nlm.nih.gov/ VEGAS: http://gump.qimr.edu.au/VEGAS/

IMPUTE2: http://mathgen.stats.ox.ac.uk/impute/impute_v2.html#home

## SUPPLEMENTARY FIGURES AND TABLES





## Abbreviations

SNP: single nucleotide polymorphism
GWAS: genome-wide association study
RCC: renal cell carcinoma
GSEA: gene set enrichment analysis
VEGAS: Versatile Gene-Based Association Study
eQTL: Expression Quantitative Trait Locus
