# Supplementary material for: Multilevel-analysis identify a cis-expression quantitative trait locus associated with risk of renal cell carcinoma
Source: Oncotarget. 2015 Feb 25;6(6):4097–109. doi: 10.18632/oncotarget.3001 (PMC4414175; doi:10.18632/oncotarget.3001)
Supplement: Supplementary file 1 [file oncotarget-06-4097-s001.pdf]

SUPPLEMENTARY FIGURES AND TABLES

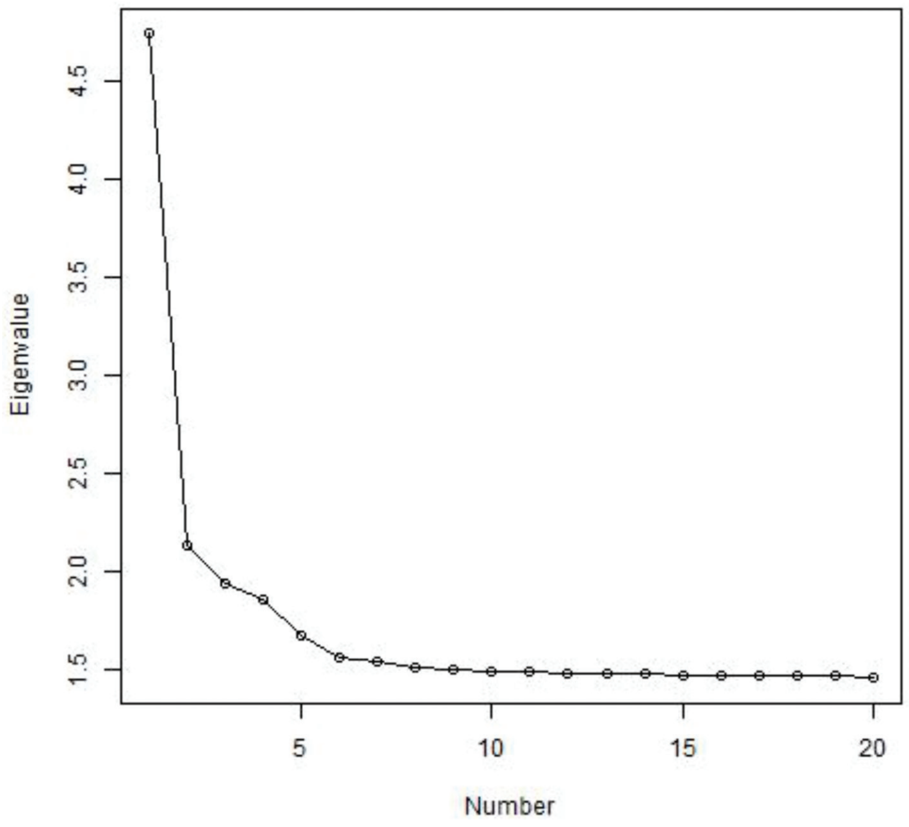

Supplementary Figure S1: Scree plot of principal components in RCC GWAS.

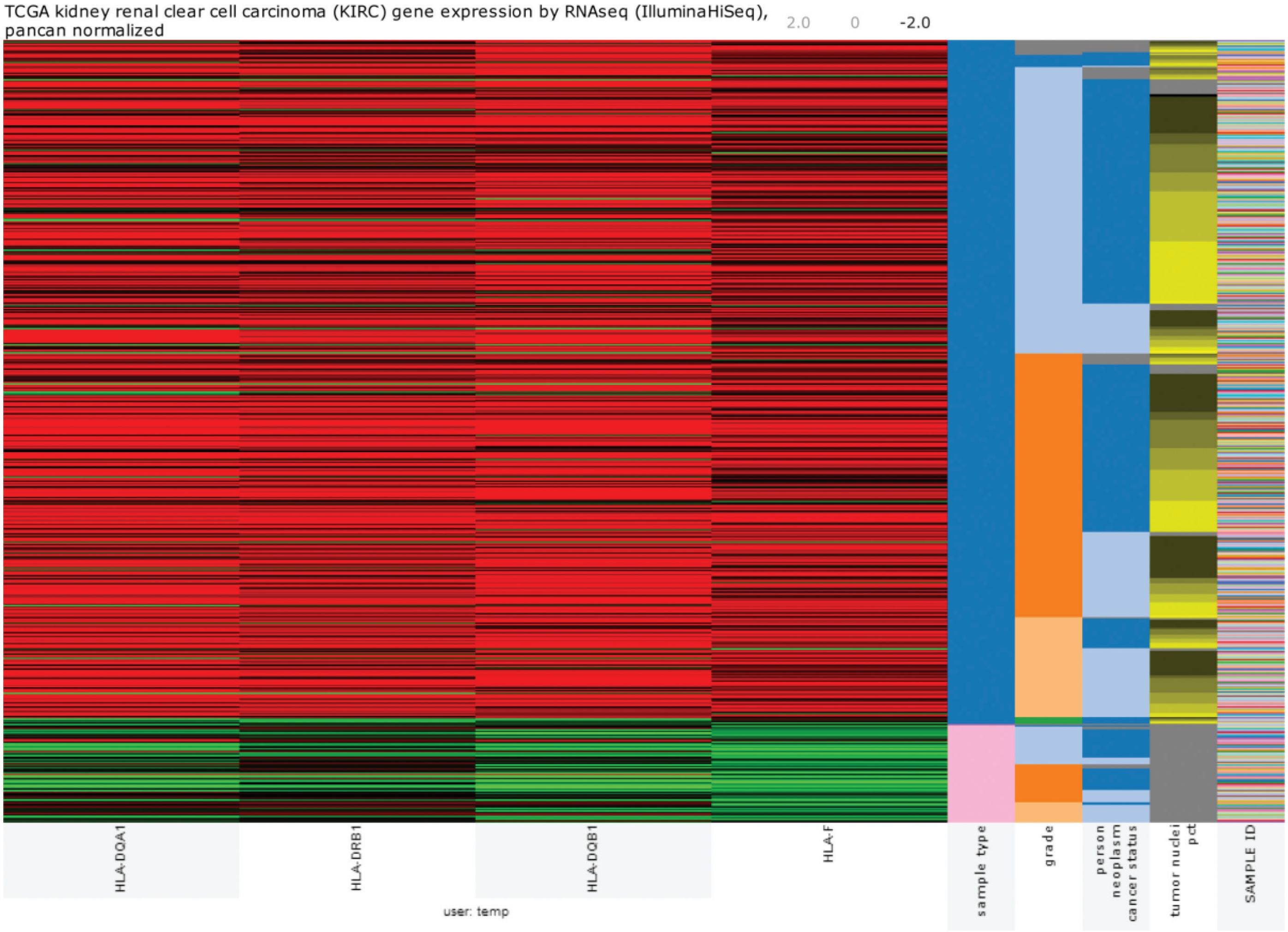

Supplementary Figure S2: Gene expression of HLA genes in RCC adjacent normal and tumor tissues in TCGA.

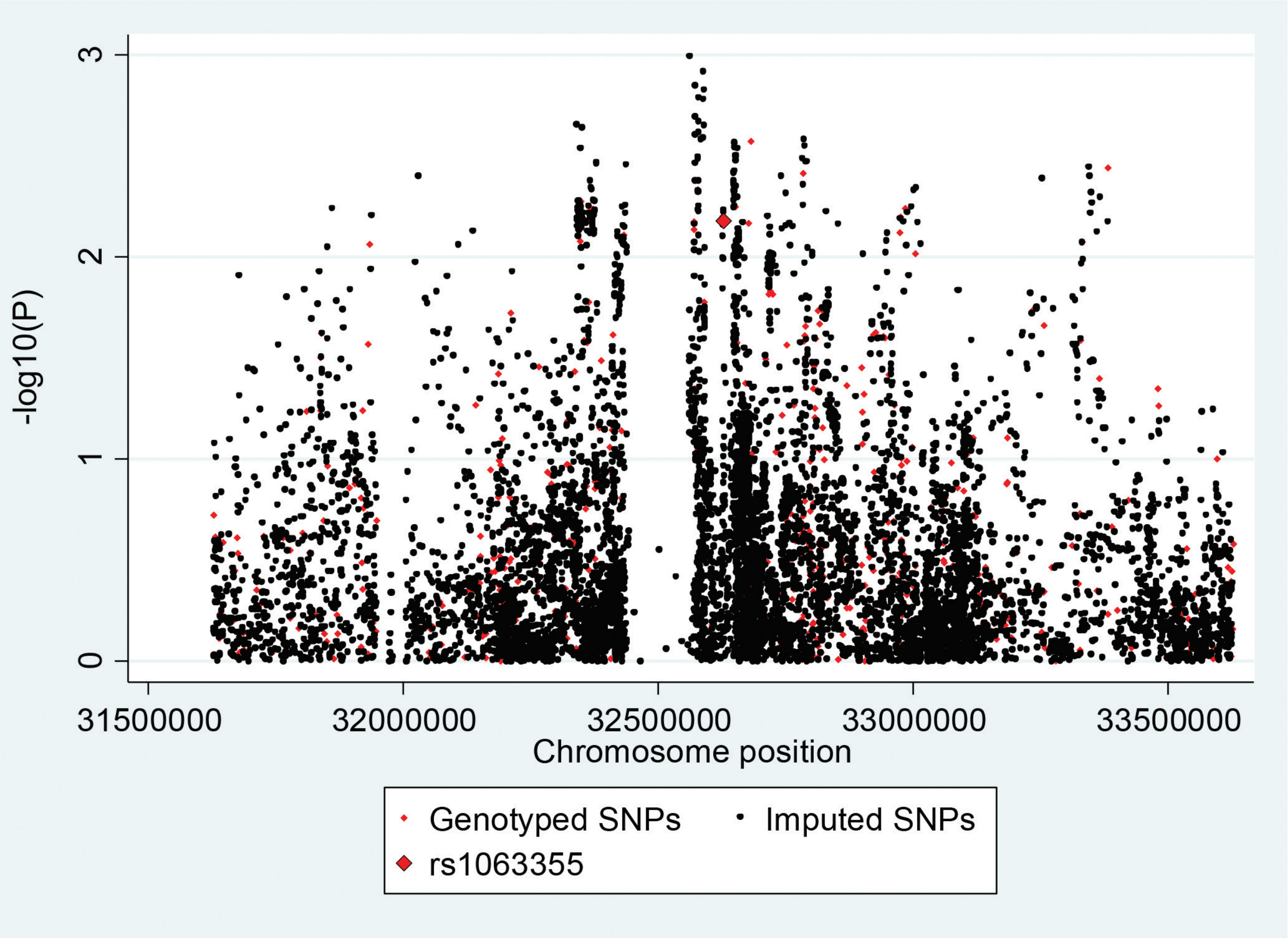

Supplementary Figure S3: Imputed location of rs1063355 within the region of +/−1 Mb.

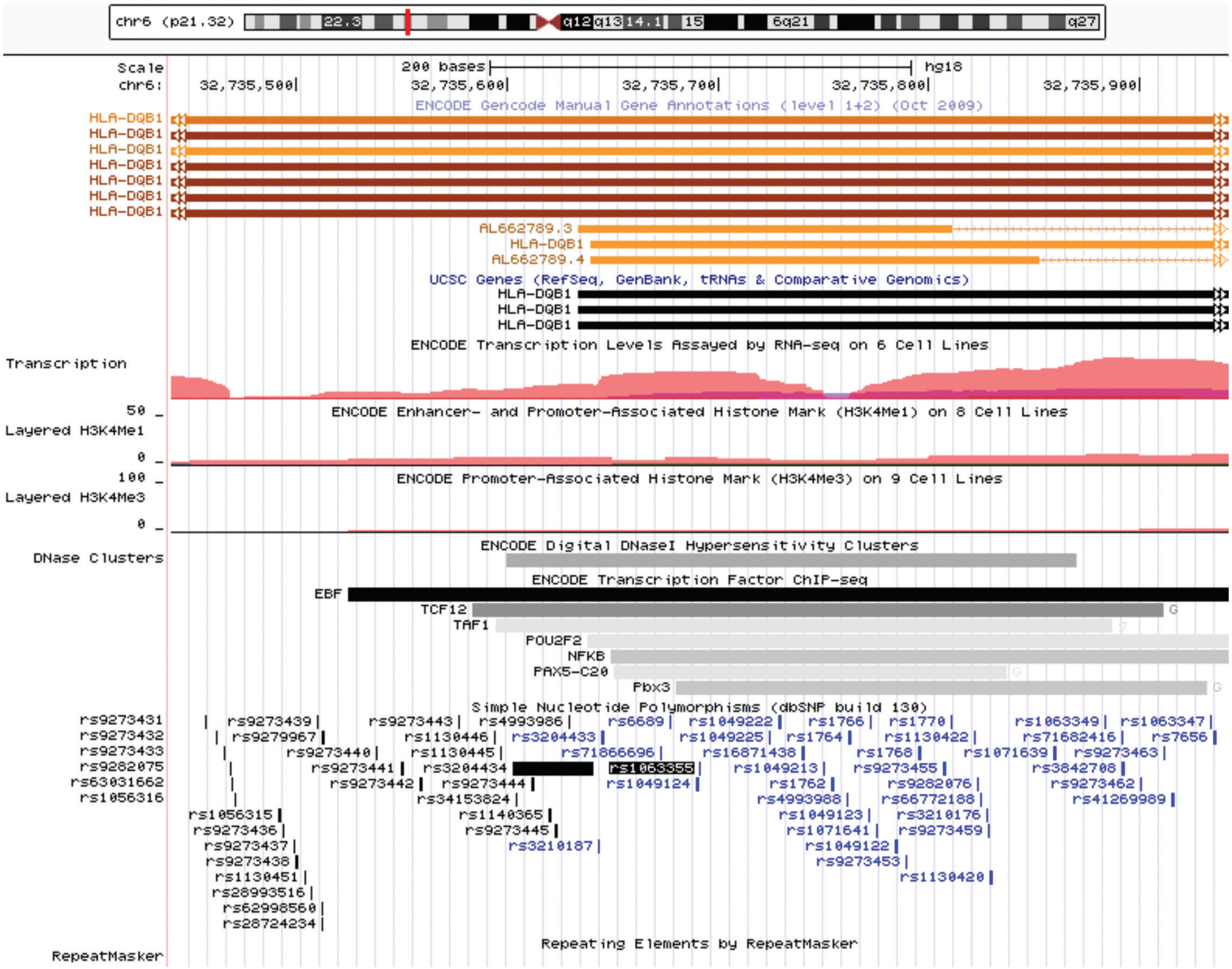

Supplementary Figure S4: The rs1063355 and transcription factor binding sites of *HLA-DQB1* at the 3'-untranslated region. This figure was downloaded from ENCODE implemented in the Genome Browser.

Supplementary Table S1: Sensitivity analyses of antigen processing and presentation pathway

Pathways	Number of genes tagged by study GWAS SNPs	*P* value		
		GenGen	SNP ratio test	ALIGATOR
Original observations	78	0.001	0.004	< 0.001
Adjustment of top 10 principal components	78	0.001	< 0.001	< 0.001

Supplementary Table S2: Gene expression comparison between paired RCC and normal tissue using oncomine database

Gene	No. of significant studies[§]	Most significant study[¶]				Average[&]	
		Author	*p* value	Fold change	Rank (%)	*p* value	Median rank
HLA-DQB1	6	Lenburg	2.96E-6	6.68	80 (1%)	6.42E-5	222
HLA-F	5	Beroukhim	3.78E-10	2.94	177 (1%)	1.00E-7	222
HLA-DQA1	4	Gumz	2.41E-9	9.77	67 (1%)	5.14E-5	746
HLA-DRB1	3	Gumz	9.41E-8	3.26	217 (2%)	0.002	1161
CREB1	2	Jones	2.49E-15	3.87	334 (3%)	1.39E-5	1231
PDIA3	2	Gumz	9.58E-5	1.60	997 (8%)	3.53E-4	1548
CTSL1	1	Yusenko	5.32E-4	2.12	1,552 (8%)	0.060	3896
PSME3	0	N.A.	N.A.	N.A.	N.A.	0.219	5440.5

[§]The number of studies with significance criteria defined as ($p < 0.001$), fold > 1.5, rank in top 10%. In total, there were 6 datasets that included the comparison of gene expression levels between RCC and normal tissue (TCGA data are not included).
[¶]Sorted by rank gene rank percentile.
[&]Average *p* value and median gene rank calculated with all 6 datasets.
N.A. Data not available since any study shows significant alteration for the gene.

Supplementary Table S3: SNPs at $P \leq 0.05$ in antigen processing and presentation pathway as defined by KEGG database

SNP	Minor allele	Odds ratio	*P* value
rs3128501	g	0.81	0.0007
rs4713240	g	0.79	0.0016
rs3128511	a	1.21	0.0020
rs2551949	g	0.79	0.0028
rs709688	a	0.69	0.0030
rs2071554	a	0.62	0.0039
rs9275141	c	0.84	0.0056
rs3130604	g	1.26	0.0058
rs2071800	a	0.72	0.0063
rs1063355	a	1.19	0.0066
rs1362126	a	0.84	0.0073
rs2516049	g	0.84	0.0073
rs2523409	g	0.84	0.0074
rs3783932	a	0.75	0.0086
rs1591033	g	1.26	0.0092
rs2230365	a	1.24	0.0105
rs3118866	a	1.16	0.0146
rs7774954	a	0.74	0.0153
rs4713600	a	0.86	0.0184
rs2071477	g	0.86	0.0184
rs2071540	a	1.16	0.0185
rs2229092	c	1.33	0.0190
rs938486	g	1.16	0.0215
rs3130257	a	1.24	0.0218
rs2517912	a	0.87	0.0219
rs3118839	a	0.87	0.0238
rs2844482	a	1.20	0.0240
rs3135029	a	1.26	0.0242
rs2239804	g	0.87	0.0243
rs2523399	g	1.15	0.0245
rs2857106	g	1.19	0.0245
rs2857107	a	0.80	0.0260
rs3093662	g	0.77	0.0277
rs2395163	g	0.85	0.0325
rs4947324	a	0.81	0.0327
rs324148	a	0.86	0.0330

(*Continued*)

SNP	Minor allele	Odds ratio	*P* value
rs1632948	g	1.13	0.0359
rs2394160	g	0.88	0.0391
rs1485591	c	0.83	0.0396
rs15251	a	0.87	0.0423
rs2429657	g	1.16	0.0437
rs3819721	a	0.87	0.0449
rs10811537	g	0.86	0.0455
rs6924102	g	0.88	0.0457
rs2394180	g	1.20	0.0459
rs2596551	c	0.85	0.0461
rs151719	g	1.15	0.0476
rs1611149	a	1.13	0.0477

All SNPs were tested in an additive model and adjusted for age (5-year intervals) and sex.

Supplementary Table S4: Function annotations of rs1063355 and SNPs in LD ($R^2 > 0.8$) using ENCODE data

Supplementary Table S5: eQTL analysis for SNPs in 3'UTR of HLA-DQB1

SNP	Source	GWAS P	R^{2*}	Spearman's Rho in normal	Correlation P in normal	Spearman's Rho in tumor	Correlation P in tumor
rs1063345	Imputed	5.84E-03	0.99	−0.652	7.00E-07	−0.688	9.16E-08
rs9273417	Imputed	6.47E-03	1	−0.618	2.21E-06	−0.612	3.03E-06
rs1063355	Genotyped	6.64E-03	N.A	−0.588	5.60E-06	−0.588	5.73E-06
rs9273415	Imputed	6.92E-03	1	−0.618	2.21E-06	−0.612	3.03E-06
rs9273443	Imputed	7.85E-03	1	−0.588	5.60E-06	−0.588	5.73E-06
rs77762292	Imputed	6.46E-02	0.39	−0.360	1.41E-02	−0.392	7.03E-03
rs113129258	Imputed	1.08E-01	0.24	−0.411	4.98E-03	−0.332	2.59E-02
rs74636670	Imputed	1.30E-01	0.24	−0.411	4.98E-03	−0.332	2.59E-02
rs1049222	Imputed	1.74E-01	0.25	−0.369	1.06E-02	−0.342	1.85E-02
rs16871438	Imputed	1.74E-01	0.25	−0.369	1.06E-02	−0.342	1.85E-02
rs34153824	Imputed	1.76E-01	0.25	−0.369	1.06E-02	−0.342	1.85E-02
rs78083269	Imputed	1.76E-01	0.25	−0.369	1.06E-02	−0.342	1.85E-02
rs1049122	Imputed	1.78E-01	0.25	−0.369	1.06E-02	−0.342	1.85E-02
rs1049225	Imputed	2.05E-01	0.48	−0.349	1.74E-02	−0.354	1.57E-02
rs1130446	Imputed	2.07E-01	0.22	−0.436	1.72E-03	−0.410	3.43E-03
rs1130455	Imputed	2.40E-01	0.26	−0.382	8.76E-03	−0.369	1.15E-02
rs117396570	Imputed	3.33E-01	0.03	−0.152	2.92E-01	0.035	8.07E-01
rs114319354	Imputed	3.44E-01	0.03	−0.152	2.92E-01	0.035	8.07E-01
rs28703037	Imputed	7.21E-01	0.07	0.051	7.28E-01	−0.124	3.96E-01

*Pairwise R2 between rs1063355 and other SNPs.
^Estimated with respect to the minor allele.
